# Comparison of post-operative outcomes after open or closed surgical techniques to stabilize metacarpal and metatarsal fractures in dogs and cats

**DOI:** 10.1186/s12917-022-03404-3

**Published:** 2022-08-04

**Authors:** Gabriel Carbonell Rosselló, Jasmin Carmel, Matthew Pead, Victor Vidal Lacosta, Pilar Lafuente

**Affiliations:** 1 Hospital Veterinario del Mar, Carrer de la Marina 69, 08005 Barcelona, Spain; 2 La Merced Veterinary Specialists, Partida Colari, 5E, 03710 Calpe, Alicante, Spain; 3 Shrubbery Veterinary Goup, New Ash Green, 5 the link, New Ash Green, Longfield, DA3 8HG UK; 4 Dept. Clinical Sciences and Services, Royal Veterinary College, London, 4 Royal College St, London, NW1 OUT UK; 5 UNIR-Universidad Internacional de La Rioja, Av. de la Paz 137, 26006 Logroño, La Rioja Spain

**Keywords:** Dog, Cat, Fracture, Metacarpus, Metatarsus, Surgery, Outcome

## Abstract

**Backfround:**

Treatment options for metacarpal/metatarsal fractures include conservative and surgical management. The aim of this study is to determine whether there is any significant difference in healing and complication rates, between open and closed treatment. Medical records of dogs and cats with metacarpal/metatarsal fractures with complete follow-up were retrospectively reviewed. Patients were allocated in two groups: open or closed stabilization. Minor and major complications were recorded and compared. Fracture healing was classified as good, delayed and non-union, and it was statistically compared.

**Results:**

Sixty-three patients (35 dogs and 28 cats) were included. Thirty-one were treated with an open approach and 32 by a closed stabilization. Regarding fracture healing a significantly higher proportion of delayed healing/non-union was found in the closed group (12/32 vs 2/31). Regarding postoperative complications, a significantly higher number of animals in the open group did not develop any complications (12/31 vs 3/32). A significantly higher proportion of minor complications were reported in the closed group (27/32 vs 12/31). However, a higher number of major complications was reported in the open group (7/31 vs 2/32) although this was not statistically significant. Fracture malalignment was significantly more prevalent in patients undergoing closed stabilization (11/32 vs 2/31).

**Conclusion:**

According to the results, better healing, fracture alignment and a lower complication rate are found when fractures are stabilised with an open technique. However, other factors such as configuration of the fracture, soft tissue involvement, patient´s character and client´s situation would also need to be taken into account in the decision of stabilization technique.

## Background

Metacarpal (MC) and metatarsal (MT) fractures are reported to occur with an incidence of up to 5–12% of all fractures in dogs and 3% in cats [1–6]. They are usually a result of trauma, most frequently road traffic accidents and falls [5–7], and most commonly affect the mid- or distal diaphysis of MCs, and the proximal region of MTs [8–10].

Conservative management has been historically recommended for minimally displaced fractures and fractures where at least one major weight-bearing bone (MCs/MTs III or IV) is undamaged [6, 9, 10]. Surgery is recommended when more than two bones are fractured, if both main weight-bearing bones are affected, or there is a proximal fracture of MCs/MTs II or V. Additionally, surgery is advised for significantly displaced fractures, open fractures, infected fractures and those in large or working dogs [5, 6, 10–12].

Various surgical techniques have been described [3, 5, 6, 11–17]. Open techniques consist of intramedullary (IM) pin fixation, internal fixation with bone plates, tension-band wire fixation, and lag screws. Closed techniques include several configurations of External Skeletal Fixators (ESFs). [6, 18, 19]

ESFs are the preferred technique for comminuted and/or for open fractures, where “biological osteosynthesis” is desired for preservation of blood supply and reduction of contamination [20–22]. Closed reduction decreases tissue trauma and infection risk at the fracture site by eliminating the soft tissue incision and approach [17, 22, 23]. ESFs provide a less rigid fixation than internal fixation and may allow micromotion at the fracture site, which has been shown to be important in stimulating blood flow and callus formation [23]. However, any further increase in motion at the fracture site will exceed the strain limits of healing tissues, leading to fibrous tissue formation and an eventual non-union. [22, 23] Surgeons must find a balance between acceptable stability for fracture healing and adequate flexibility to allow stimulatory micromovement. Epoxy putty external skeletal fixation has been described as a successful technique for the management of multiple MC and MT fractures in 11 dogs and 11 cats when a good alignment is achieved [24].

Circular external skeletal fixation has also been described as an option to treat multiple MT and MC fractures in three dogs [25]. The main advantage of using a circular fixator construct is the small-diameter fixation wires. Despite local inflammation and drainage tract from the wires were seen in all cases, none of the dogs had residual lameness in the follow up period [25].

An external skeletal traction device for distal fractures has been described in eleven dogs as an option for closed treatment [26]. The majority of the fractures had an improvement in both alignment and apposition postoperatively. In multiple metatarsal or metacarpal fractures that are not amenable to internal fixation or external coaptation, the traction- ESF device can provide a valuable alternative in fracture management. This is especially beneficial if the wounds are infected and require frequent attention and dressing [26].

Open fixation methods can disrupt blood supply at the fracture sites to a greater extent than closed methods, [21] which could lead to increased risk of delayed healing, non-union and/or infection [7, 22]. For simple or mildly comminuted fractures, internal fixation generally provides better alignment, [25] and the surgeon must decide whether this advantage outweighs the detriment of disturbing the healing environment. Intramedullary pins can also achieve satisfactory stabilisation with or without additional stabilization [10, 12, 27]. Intramedullary pins can be applied in a normograde or retrograde (Dowel pinning) fashion. Normograde application of the k-wires/pins usually requires the creation of a dorsal slot in the distal aspect of the bone to allow the pin to slide into the medullary cavity and not getting driven into the opposite cortex. A “dowel” intramedullary pin technique has been described in cats and avoids both joint penetration and drilling of a slot in the dorsal MT or MC cortex [25].

The veterinary cuttable plate is a traditional implant for the open stabilization of metacarpal/tarsal fractures. Other commonly used implants are locking plates of appropriate size. A minimally invasive approach to the repair of meta-bone fractures represents a viable option with several benefits related to the preservation of the local biology. Fractures of the body of meta-bones III and IV can be approached by creating 1 or 2 small skin incisions proximally and distally to the fractured area [23].

ESFs can be used together with intramedullary pins, [7, 9] which allows more accurate anatomical alignment of fracture fragments than closed techniques alone. This is the case of the Spider external fixator. The surgical technique involves normograde or retrograde intramedullary pin placement into the fractured MT/MC bones and transverse pin placement at the base of the MT/MCs or tarsal/carpal bones. The distal pin ends are contoured dorsally in epoxy resin and implants maintained until fracture union. Pin penetration of MT-phalangeal or MC-phalangeal joints may cause morbidity and requires further study [9]. Distal application of the pin without open approach to the fracture site can limit damage to vascularity, thereby retaining more biological potential compared to other open repair techniques [7, 9].

There have been some studies comparing the efficacy of conservative versus surgical management of MC and MT fractures in dogs and cats, [10, 13] however, to the authors knowledge, there are no studies describing differences between outcomes of open versus closed surgical repair of metatarsal or metacarpal fractures.

The objective of this study was to gather data on healing and complications from patients with metatarsal or metacarpal fractures, and test the hypothesis that there is a difference in these parameters between those treated with an open surgical approach as opposed to a closed surgical approach.

## Results

Sixty-three patients with metatarsal and/or metacarpal fractures were included in this study. Patients were categorised into two fracture repair groups: open (31 patients) and closed (32 patients). Patients also consisted of 35 dogs (19 open, 16 closed) and 28 cats (12 open, 16 closed). Patient age ranged from 2 months to 13 years (mean of 4.6 years ± 2.91 for the open group, and 4.29 years ± 3.87 for the closed group). No significant differences were found between groups. In the open group 4 animals were female entire, 9 were female neutered, 5 male entire and 13 males neutered. In the closed group 1 animal was female entire, 11 females neutered, 8 male entire and 12 males neutered. Tables 1 and 2 outline the nature of the fractures found in each repair group. All of the single metacarpal/metatarsal fractures occurred in the 2^nd^ or 5^th^ bone, causing instability, and therefore surgical stabilization were elected.

**Table 1:** Recorded details of fractures in the open and closed repair groups

Fracture details	OPEN	CLOSED
Single MT/MC bone fracture	16	7
Multiple MT/MC bone fractures	15	25
Comminuted fracture	7	4
Ligament injury / joint Instability	17	8
Concurrent limb fractures	5	7

**Table 2:** Recorded causes of fractures in the open and the closed repairs groups

Cause of fracture/s	OPEN	CLOSED
Road traffic accident	5	10
Falling or jumping	11	5
Running	6	1
Object struck foot	1	3
Dog bite	2	1
Foot trapped	1	0
Unknown	5	12

Time between trauma and surgery ranged from 1 to 28 days for the open group (mean 4.43 ± 4.93 days) and 1 to 21 days for the closed group (mean 4.26 ± 5.17 days) No significant differences were found between groups. For 11 patients, time to surgery was not apparent in the clinical notes.

Open stabilization techniques applied included plates and screws, (DCP; VCP, arthrodesis plate), intramedullary (IM) pins (normograde, Dowel pinning), IM pins with ESF. Table 3 outlines the number of dogs for the different fixation methods used in the open repair group.

**Table 3:** Type of fixation used in the open repair group

Fixation method	Number of patients
Dynamic compression plate	7
Veterinary cuttable plate	3
Arthrodesis plate	6
Other plates	4
IM pins (normograde)	2
IM pins (Dowel)	5
IM pins with ESF	4

Closed surgical techniques used included external skeletal fixation (32 patients).

Twenty-three patients in the open group (23/31) were supported with external coaptation during their post-operative period (14 splinted/cast, 9 non-splinted). Eighteen patients in the closed group (18/32) had external coaptation applied (5 splinted/cast, 13 non-splinted).

**Fig. 1:**
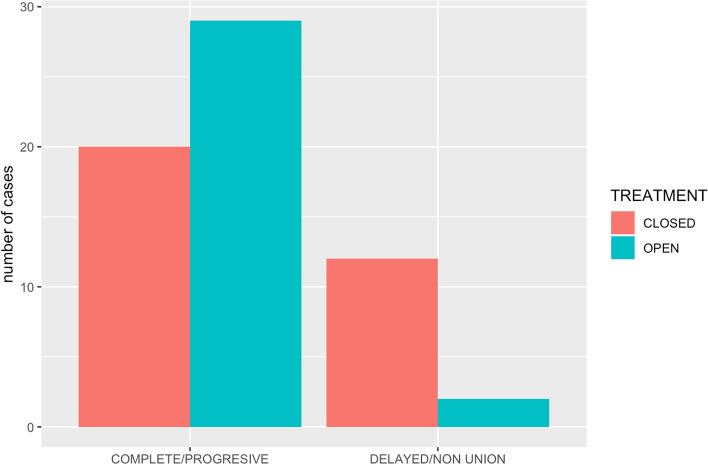
The proportion of patients allocated to each healing category, in open and closed repair groups

Regarding healing of the fracture/s, 29 patients in the open group (29/31) had good healing and 2 showed delayed healing/non-union (2/31). In the closed repair group, 20 patients showed good healing of the fractures (20/32), and 12 presented delayed healing/non-union (12/32) (Fig. 1). Statistical analysis revealed a significant difference in fracture healing between groups, (*p* = 0.008) with a significantly higher proportion of delayed healing/non-union in the closed group (37,5%) than in the open group (6,4%).

Major complications recorded included iatrogenic fracture, failure of surgical implants that required revision surgery or implant related problems that required explantation.

Minor complications recorded included malalignment/malunion and non-union not requiring surgery, delayed union, mild-moderate persistent lameness, infection, altered gait, muscular atrophy, pain, metatarsal/metacarpal resorption, metatarsal/metacarpal synostosis, limb deformities (e.g. tarsal valgus), bandage sores and vomiting.

**Fig. 2:**
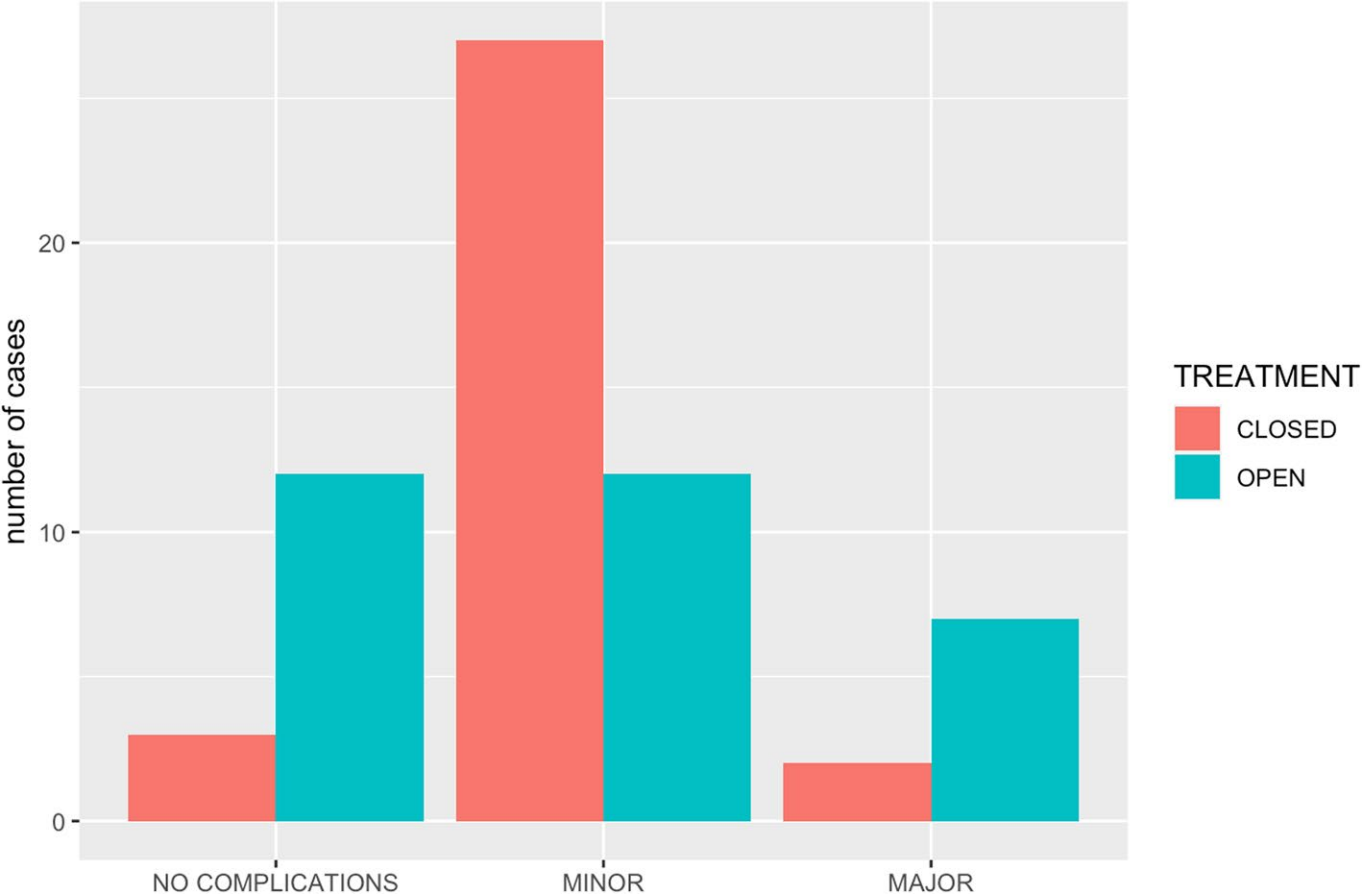
Comparing the number of repeat visits for open and closed repair techniques

Nineteen out of 31 patients in the open group had complications; 12 patients had minor, and 7 had major complications. In the closed group, 29 out of 32 patients had complications; 27 patients had minor complications and 2 showed major complications (Fig. 2). Analysis revealed that the differences between treatment groups was statistically significant (*p* = 0.001) with more complications in patients undergoing closed stabilization (29/32 vs 19/31). These differences were mainly due to significant differences found between groups in minor complications (*p* = 0.003). There was no statistically significant difference in major complications, but the rates of 7/31 (22.5%) for the open group and 2/32 (6.3%) may be clinically important. The majority of major complications observed in the open group were related to implant infection (5/7) or implant migration in 2/7 cases associated with a K-wire migration. Given the internal location of the implants, a revision surgery could be required in those cases. On the contrary, pin tract/implant infection with ESF is a frequent encountered complication that can generally be managed without surgery, and therefore was considered a minor complication in our study. Implant related complications that required additional surgery in the closed group (i.e. implant fracture/migration) only affected 2/32 cases, which were considered major complications.

For complications experienced by both groups, the closed group experienced a greater percentage of delayed union, malunion/mal-alignment, infection, implant failure/migration, mild-moderate persistent lameness, muscle atrophy and limb deformities. The open group had a greater percentage of revision surgery, bandage complications, and reduced range of motion of the carpus/tarsus.

**Fig. 3:**
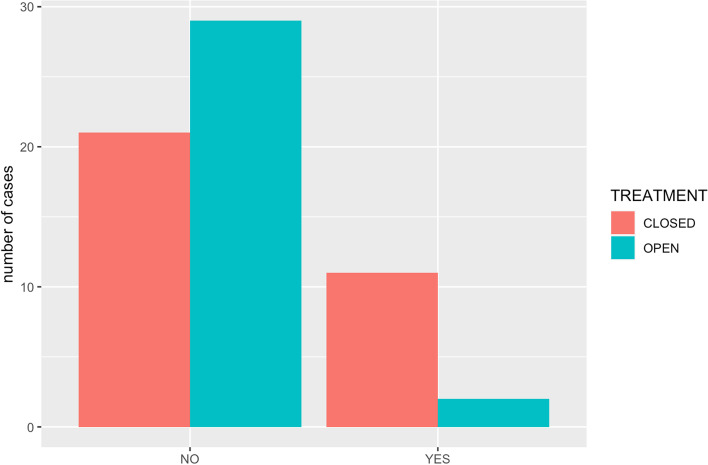
Comparing the number of complications in the open and the closed repair group

Among minor complications, malalignment was present in 11 out of 32 patients in the closed group and in 2 out of 31 patients in the open group (Fig. 3). This complication was significantly more prevalent in patients undergoing closed stabilization (34.4%) than in animals in the open group (6.4%) (*p* = 0.015).

Eight out of 23 animals developed bandage related complications in the open group and 2 out 18 in the closed group. The difference in the number of bandage complications was not found to be significant. (*p* = 0.08).

**Fig. 4:**
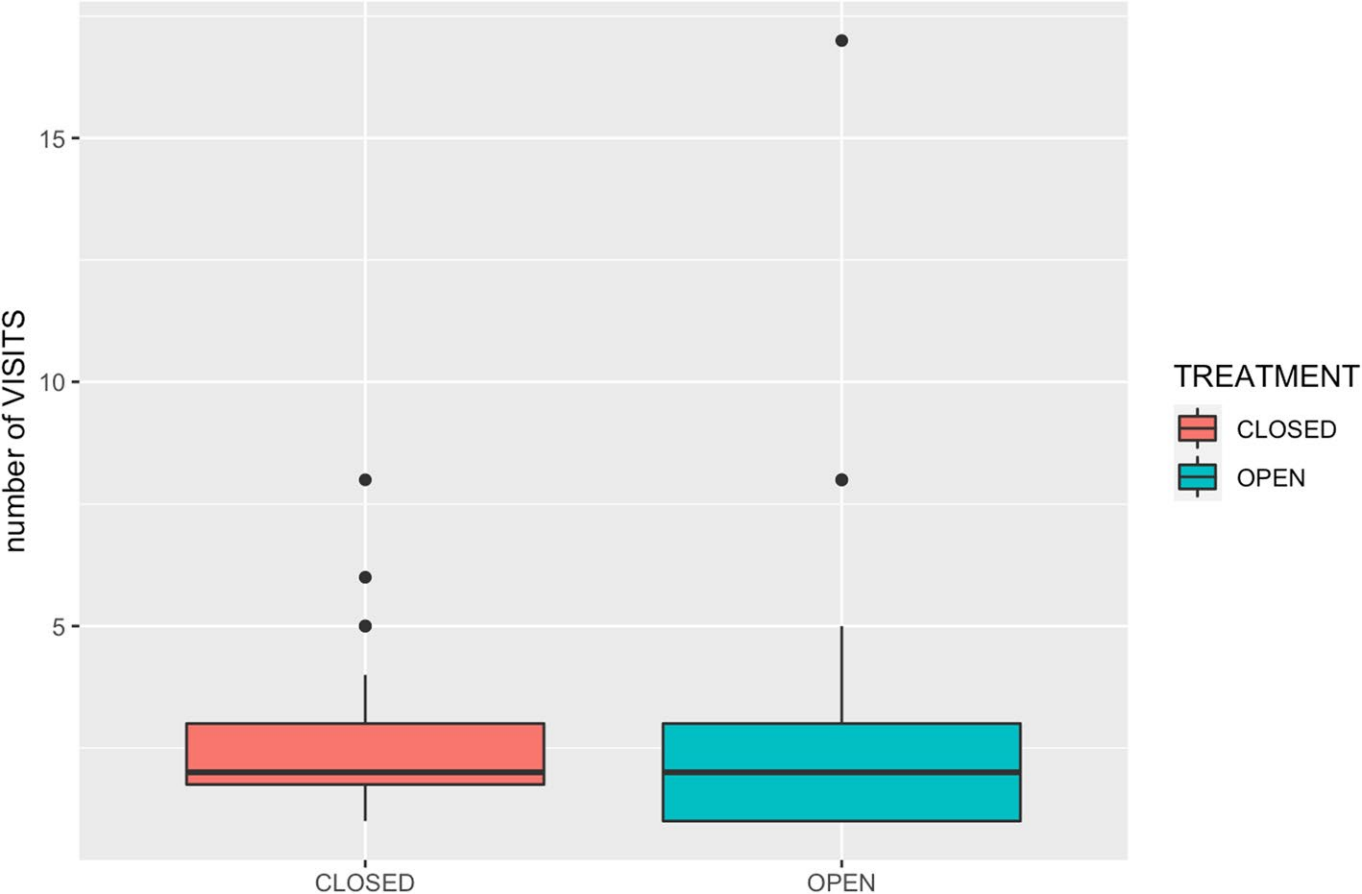
Comparing the number of cases experienced malalignment between open and close groups

The mean number of recheck visits for the open group was 2.84 (± 3.25), and for the closed group was 2.59 (± 1.64). The range was 1–17 and 1–8 for the open and the closed group, respectively. Statistical analysis revealed no significant difference (*p* = 0.706) between the number of recheck visits for open and for closed repairs (Fig. 4).

## Discussion

The objective of this study was to check if there are differences in healing and complications between the healing of metatarsal or metacarpal fractures that were treated with an open versus a closed approach. The results show that there are differences between the two types of treatment in some of the parameters that were analysed, indicating that these differences are significant, and should be taken into account when deciding on clinical treatment.

There was a statistically significant difference in fracture healing between groups (*p* = 0.008), with a higher proportion of delayed healing/non-union post-operatively (12/32) in the closed (ESF) treatment group when compared to open treatments (2/31). Although open repair disrupts the early stages of fracture healing,[6, 22] the superior alignment and reduction achieved [23], that we noted in this study (Fig. 3) may have compensated for this and allowed more rapid, complete healing than the closed techniques. However, open, multiple and comminute fractures are most commonly stabilised in a closed manner, so the inherent severity of these fractures may also be playing a role in the higher proportion of delayed healing and non-union cases in the closed group. In the study presented here most of the fractures affecting multiple MC and MT bones were managed by closed stabilization (25/40 vs 15/40). However, there were more comminuted fractured managed by an open stabilization technique (7/11) than by a closed technique (4/11).

A greater degree of malalignment is inevitable in closed techniques and in this study, it was significantly more prevalent in patients undergoing closed stabilization (34.4%) than in animals in the open group (6,5%). Malalignment could increase the risk of delayed union and non-union. In this study these were considered as minor complications since they did not require further intervention as it was described by Jackson, L et al. in 2004. Malunion and malalignment, as well as non-union, are commonly considered as major complications in long bone fractures because it causes a significant disfunction and could require revision surgery. However, in the MC and MT bones, they are frequently not clinically significant as one or more bones with an imperfect union or non-union may be supported by the other bones and form a functional pes or manus. In this study the presence of malunion/non-union in a reduced number of bones did not require further intervention.

There was a significantly larger proportion of animals in the closed group with complications compared with the open group when the major and minor complications were grouped together. In the closed group 29/32 of the patients experienced complications while 19/31 had complications in the open group. Most of these complications were minor and did not require further intervention. The difference between major complications in the open group 7/31 and the closed group 2/32 was not statistically significant, but the small number of these complications makes any statistical significance harder to detect. However, the fact that 22.5% of the open group patients required a repeat surgery, mainly because of implant infection (5/7) or K-wire migration (2/7), while only 6.3% of the closed group patients experienced a major complication and these could be dealt with by changes in the fixator, is of clinical interest.

The presence of complications often generates further hospital visits but in this study, there was no statistical difference detected in post-operative visits between the groups. It may be that studies with a larger sample size would help to make the relationship of treatment, complications and hospital visits clearer, but the fact that minor complications can often be treated by the client with telephone advice or the referring veterinary surgeon may mean that the hospital visit numbers is not a very clear measure of the difficulties engendered by complications.

Bandage complications were not significantly different between the open group compared to the closed group. What is clear from these results is that both the closed and open treatment groups had a considerable number of complications and when treating these fractures this should be taken into account.

Closed treatment in this study relied solely on the ESF. There is a logic to using the ESF in the MC/MT areas. Soft tissue coverage is minimal and the bones themselves relatively small, so a minimally invasive system that allows for a varied size of small implants and has been shown to preserve vascularisation, [21] is a logical choice. However, the inevitable consequence of a closed reduction in such small bones are compromises on alignment, when compared to open reduction, and this would contribute to some of the effects on bone union seen here. In this study there was an increased rate of infection in the closed group (ESF) and this is similar to other reports of ESF fixation. It is well known that ESF fixation is associated with a relatively high risk of minor complications, especially pin tract infection [28, 29] and a prevalence of 37% of pin tract infection in animals where External Skeletal Fixation has been applied [29] has been reported. Pin tract infection is an important factor in the failure and migration of external fixation implants [30, 31]. It is likely that this phenomenon was related to the implant failure/migration seen in this group.

The variety of fixation techniques used in the open group also generated clinically significant numbers of complications (19/31). There were fewer minor complications, so significant numbers of these patients would have had a less troubled recovery, and the minor complications were similar in nature to those in the closed group. Some of these, such as low-grade infection could be treated with medical therapy, and some like delayed union or malalignment did not have a major impact on the recovery of the patient. The significant difference between closed and open fixation is the surgical exposure, potential compromise of vasculature and soft tissue integrity, coupled with the persistence of implants at the site of the fracture. These factors are linked to wound breakdown, infection and the potential for poor bone healing and implant failure. In the open group 7/31 (22.5%) experienced complications of this nature requiring revision surgery. This is a much higher rate than would be expected for long-bone fixation [28–31] and demonstrates the vulnerability of this site to these problems.

Time to full healing was another parameter the authors wished to measure to further define the quality of healing in each repair group. However, due to incomplete data, radiographic union and/or resolution of lameness could not be reached for every patient, and therefore an endpoint could not be ascertained. Further studies that compare time to radiographic union/resolution of lameness could be helpful to further compare outcomes after open and closed stabilization techniques in the management of metacarpal and metatarsal fractures in dogs and cats.

This study is not exempt of limitations. As a retrospective study, this investigation was entirely dependent on the completeness of clinical records. Additionally, factors such as breed and age of patients could not be controlled. Age has been shown to have an impact on the outcome of fracture healing, and both size and age play a role in driving the decision to make an open or a closed approach [11, 12, 32]. In this study, no patients under 1 year of age exhibited delayed healing or non-union.

## Conslusions

In the treatment of any limb fracture the overall goal is to restore function of the limb. In the metacarpal/metatarsal area restoring limb function is often more about ensuring that the patient can position the paw on the ground correctly and providing a stable manus for load transmission, than achieving perfect reduction and healing in any one bone. This study shows that choosing between closed (ESF) fixation and open reduction and internal fixation for metacarpal and metatarsal fractures is likely to mean significant differences in the complications experienced, and the progress of healing. It also shows that there is a high risk of complications and a need for careful post-operative care irrespective of the method of repair chosen. Both types of repair appear to lead to a successful long-term outcome in most cases, so the ability of a particular patient to cope with types of complications and difficulties outlined in this study may be the most significant factor in the choice of repair method. These findings may assist clinicians in their decision-making process for the management of metacarpal /metatarsal fractures in dogs and cats.

## Methods

The medical records of dogs and cats presented to the Royal Veterinary College Queen Mother Hospital for Animals (QMHA) with metatarsal and metacarpal fractures were retrospectively reviewed. All methods were performed in accordance with the relevant guidelines and regulations. Relevant cases were found using VetMine, the built-in search engine of the QMHA’s computarised database for patient records (CRIS). Patients were recruited if they had a relevant fracture confirmed via diagnostic imaging, and allocated into groups based on whether their fractures were repaired with an open or a closed technique. Patients treated with a combination of open and closed techniques were placed in the “open” group. Cases managed with an External Skeletal Fixator were placed in the “closed” group unless clinical notes provided evidence that an open approach was made. Exclusions included patients who did not return to the QMHA post-treatment, patients who had no more recorded information post-operatively, patients whose fractures had already been stabilised by the referring vet, fractures that occurred as complications of another surgery and pathological fractures.

For each patient the following data was recorded: signalment, type of fracture, cause of fracture, management and nature of surgery performed, number of recheck visits, radiographic evaluations of the fracture post-stabilization, and type and number of post-operative complications.

Patients were further allocated into three groups based on their healing status upon their scheduled post-operative re-examinations: good healing, delayed healing and non-union. Patients were placed in the first group when progressive fracture healing and/or callus formation, or radiographic union of the metatarsal/metacarpal fractures was obtained in the time expected for that fracture to heal. “Union” was defined as radiological presence of bridging callus in 3 out of 4 cortices on orthogonal radiographic views [32–34]. Patients were included in the delayed healing group if they did not have a union at their last scheduled re-check radiographs but fracture healing was progressing although at a slower speed. Patients were included in the non-union group when they required intervention to stimulate fracture healing e.g. repeat surgery, or when radiographic non-union was described.

Complications were defined as any undesirable outcome associated with treatment and were categorized as minor (managed non-surgically) or major (managed surgically).

Descriptive statistics were calculated for several variables. Chi-square tests were used to examine associations between treatment groups and various factors (healing, complications, malalignment, bandage, and number of visits). Statistical software and a spreadsheet software were used for the analysis. *P* values < 0.05 were accepted as significant 

## Data Availability

The datasets used and/or analyzed during the current study are available from the corresponding author on reasonable request.

## Abbreviations

CRIS: Clinical records information system
ESF: External skeletal fixator
IM: Intramedullary
MC: Metacarpal
MT: Metatarsal
VS: Versus
QMHA: Queen mother hospital for animals
ROM: Range of motion
SSRERB: Social science research ethical review board
DCP: Dynamic compression plate
VCP: Veterinary cuttable plate

## References

[1] Minar M, Hwang Y, Park M (2013). Retrospective study on fractures in dogs. J Biomed Res.

[2] Failing K, Matis U, Kornmayer M (2014). Long-term prognosis of metacarpal and metatarsal fractures in dogs. Vet Comp Orthopaed.

[3] Kornmayer M, Matis U, Zahn K (2007). ‘Dowel’ pinning for feline metacarpal and metatarsal fractures. Vet Comp Orthopaed.

[4] Phillips I (1979). A survey of bone fractures in the dog and cat. J Small Anim Pract.

[5] Muir P, Norris J (1997). Metacarpal and metatarsal fractures in dogs. J Small Anim Pract.

[6] Gemmill T, Clements D. BSAVA Manual of Canine and Feline Fracture Repair and Management. 2nd ed. Wiley. 2016. p. 342–346.

[7] Harari J (2002). Treatments for feline long bone fractures. Vet Clin N Am-Small.

[8] Emmerson T, Moores A, Pead M (2008). Epoxy putty external skeletal fixation for fractures of the four main metacarpal and metatarsal bones in cats and dogs. Vet Comp Orthopaed.

[9] Fitzpatrick N, Riordan J, Smith T (2011). Combined Intramedullary and External Skeletal Fixation of Metatarsal and Metacarpal Fractures in 12 Dogs and 19 Cats. Vet Surg.

[10] Gomaa M, El Seddawy F, Behery A (2016). Different modalities of metacarpal fracture fixation in mongrel dogs: An experimental study. Adv Anim Vet Sci.

[11] Wernham B, Roush J (2010). Metacarpal and metatarsal fractures in dogs. Compendium.

[12] Decamp C, Johnston S, Déjardin L, Schaefer S. Fractures and Other Orthopedic conditions of the Carpus, Metacarpus, and Phalanges. In Brinker, Piermattei and Flo’s handbook of small animal orthopedics and fracture repair. 5th ed. Elsevier. 2016. pp. 418–425.

[13] Bernasconi C, Von Werthern C (2000). Application of the Maxillofacial Mini-plate Compact 1.0 in the Fracture Repair of 12 Cats/2 Dogs. Vet Comp Orthopaed.

[14] Benedetti L, Berry K, Bloomberg M (1986). A technique for intramedullary pinning of metatarsals and metacarpals in cats and dogs. J Am Anim Hosp Assoc..

[15] Degasperi B, Gradner G, Dupré G (2007). Intramedullary Pinning of Metacarpal and Metatarsal Fractures in Cats Using a Simple Distraction Technique. Vet Surg.

[16] Roe S (1992). Classification and Nomenclature of External Fixators. Vet Clin N Am-Small.

[17] Howe-Smith R, Kapatkin A, Shofer F (2000). Conservative versus surgical treatment of metacarpal and metatarsal fractures in dogs. Vet Comp Orthopaed.

[18] Harasen G (2012). Orthopedic hardware and equipment for the beginner. Part 3: External skeletal fixators. Can Vet J.

[19] Marsell R, Einhorn T (2011). The biology of fracture healing. Injury.

[20] Aron D, Palmer R, Johnson A (1995). Biologic strategies and a balanced concept for repair of highly comminuted long bone fractures. Compendium.

[21] Palmer R (1999). Biological Osteosynthesis. Vet Clin N Am-Small.

[22] Zahn K, Kornmayer M, Matis U (2007). ‘Dowel’ pinning for feline metacarpal and metatarsal fractures. Vet Comp Orthopaed.

[23] Piras A, Guerrero T (2012). Minimally Invasive Repair of Meta-bones. Vet Clin N Am-Small.

[24] Harari J, Seguin B, Bebchuk T (1996). Closed Repair of Tibial and Radial Fractures with External Skeletal Fixation. Compendium.

[25] Seibert RL, Lewis DD, Coomer AR (2011). Stabilisation of metacarpal or metatarsal fractures in three dogs, using circular external skeletal fixation. N Z Vet J.

[26] Risselada M, Verleyen  P, Van Bree H, Verhoeven G (2007). The use of an external skeletal traction device for distal fractures in the dog. A clinical case series of 11 patients. Vet Comp Orthopaed.

[27] Dudley M, Johnson AL, Olmstead M (1997). Open reduction and bone plate stabilisation, compared with closed reduction and external fixation, for treatment of comminuted tibial fractures: 47 cases (1980–1995) in dogs. J Am Vet Med Assoc.

[28] Perry KL (2015). Bruce M, Impact of fixation method on postoperative complication rate following surgical stabilization of diaphyseal tibial fractures in cats. Vet Comp Orthopaed.

[29] Beever LJ, Giles K, Meeson R (2018). L, Postoperative Complications Associated with External Skeletal Fixators in Dogs. Vet Comp Orthopaed.

[30] Jackson L, Pacchiana P (2004). Common complications of fracture repair. Clin Tech Small Anim Pract.

[31] Horstman C, Beale B, Conzemius M (2004). Biological Osteosynthesis Versus Traditional Anatomic Reconstruction of 20 Long-Bone Fractures Using an Interlocking Nail: 1994–2001. Vet Surg.

[32] Whelan D, Bhandari M, McKee M (2002). Interobserver and intraobserver variation in the assessment of the healing of tibial fractures after intramedullary fixation. J Bone Joint Surg Am.

[33] Panjabi M, Walter S, Karuda M (1985). Correlations of radiographic analysis of healing fractures with strength: A statistical analysis of experimental osteotomies. J Orthop Res.

[34] Salih S, Blakey C, Chan D (2015). The callus fracture sign: a radiological predictor of progression to hypertrophic non-union in diaphyseal tibial fractures. Strategies Trauma Limb Reconst.

